# Supplementary material for: Learning curve and surgical outcome of robotic assisted colorectal surgery with ERAS program
Source: Sci Rep. 2022 Nov 29;12:20566. doi: 10.1038/s41598-022-24665-w (PMC9709162; doi:10.1038/s41598-022-24665-w)
Supplement: Supplementary file 1 — Supplementary Information 1. [file 41598_2022_24665_MOESM1_ESM.zip › LSC Raw data-Ñ[▒K/057/OP_ñw╝╨░O▒Kñσ.pdf]

正式摘要(電子簽章：000000181976314)

W55 / W55

臺中榮民總醫院

Operation Note

[redacted]

- 手術日期：2019/12/14
- 手術醫師：蔣鋒帆，CR 林昌霖，R 陳怡豪
- Anesthesia：GE
- Position：Lithotomy
- Aseptic agents：Betadine Alcohol，Betadine Aqua

Diagnosis

- Pre-operative diagnosis : CA OF S-COLON FOR LAPA-OP
- Post-operative diagnosis : upper rectal cancer, s/p laparoscopic Low Anterior resection on 20191214, cT1N0M0.

Surgical Indication

- For curative resection

Operation

- 1. Laparoscopic low anterior resection with end-to-side anastomosis, endo-GIA 60/4.1mm*2, CEEA 33mm, re-inforcement with silk: nil, bilateral doughnuts: intact, air leak test: done, no air leak.
  2. High ligation (+): IMA above left colic artery.
  3. Cut margin: proximal 1cm, distal 20cm.
  4. Reperitonealization: nil.
  5. Take down of splenic flexure: nil.
  6. One 12Fr. Penrose drain at pelvis.

Finding

- 1. A 1x1cm ulcerative Borrmann's type I tumor was found at upper rectum, s/p clip mapping, which limited in submucosa and mucosa layer. on enlarged indurated lymph nodes at mesocolon. Clinical stage: T1N0M0.
  2. No synchronous tumor or polyp was found.
  3. There was no visible liver metastasis, and no visible peritoneal seeding.

Procedure

- General endotracheal anesthesia was induced and then the patient was placed in modified Trendelenburg lithotomy position. Preoperative antibiotics were given. A Foley catheter and nasogastric tube were placed. The abdomen and perineum was prepped and draped in standard sterile fashion. A small vertical incision was made above the umbilicus and a 12mm trocar was inserted into the peritoneal cavity by open method. Peumoperitoneum was established with CO2 inflation. Laparoscopy was inserted and then a 12mm trocar was inserted over RLQ of abdomen. Three 5mm trocars were inserted over RUQ, LUQ and LLQ of abdomen. The liver, omentum and peritoneum were inspected for evidence of metastatic disease. No metastatic disease was noted. A mass lesion was noted in the sigmoid colon. The small bowel was inspected and retracted to upward by grasper. The sigmoid colon was then held by grasper and the peritoneum overlying the mesentery was then scored with electrocautery. The inferior mesentery artery was identified, ligated above the left colic artery with endoclips, and transected, so was inferior mesentery vein. Dissection was done above the Gerota's fascia and toward the peritoneal attachments. The arcade vessels to the sigmoid colon were also divided. Dissection was done above the Gerota's fascia and toward the splenic flexure. Using electrocautery, omentum was freed from the transverse colon. The colon was then freed from its peritoneal attachments along the line of Toldt proximally from the splenic flexure

- and distally to the pelvic inlet. A linear stapler was then used to divide the rectum at least 5 cm distal to the palpable lesion. The proximal colon was carefully inspected, noted to reach easily into the pelvis, and to have an excellent blood supply. A LLQ incision wound was made. The mesenteric fat was gently cleared from the terminal 2-3 mm of the bowel ends. It was determined that a 31 mm circular stapler could be accommodated. A pursestring suture of 3-0 Prolene was placed, the anvil was inserted, and then the pursestring suture was tied. The anal canal was irrigated with distil water and then the circular stapler was introduced through the anus and pierced through the rectal stump. The proximal bowel with anvil in place was engaged with the circular stapler and the stapler was then fired. Two complete donuts were obtained and submitted to pathology. The anastomosis was tested for leaks with irrigation solution and sir introduced through the rectum and was found to be patent and intact. The fascia of midline wound was closed with interrupted sutures of No.1 Vicryl. The fascia of the 12 mm trocar was closed with 1-0 Dexon. Subcutaneous tissues were closed with interrupted 3-0 Vicryl and the skin was approximated with dermabond. The patient tolerated the procedure well and was brought to the POR in stable condition.
- Estimated blood loss: minimum
- 器械數量: 正確
- 紗布數量: 正確

Image

- PAINT
  - colon & rectum

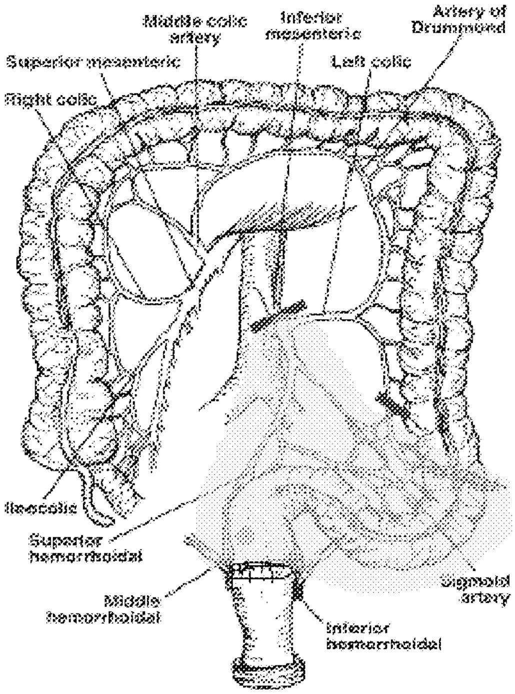

  - abdomen

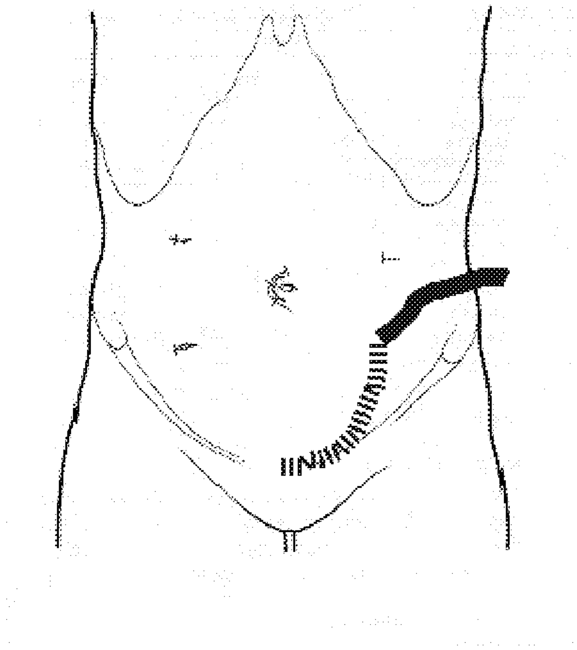

編輯主任 2062F蔣鋒帆、主治 2320B林昌霖

正式摘要(電子簽章：000000181976314)

W55 / W55

doctorRank:B
